# Longitudinal Changes of Sensorimotor Resting-State Functional Connectivity Differentiate between Patients with Thalamic Infarction and Pontine Infarction

**DOI:** 10.1155/2021/7031178

**Published:** 2021-10-08

**Authors:** Peipei Wang, Zhenxiang Zang, Miao Zhang, Yanxiang Cao, Zhilian Zhao, Yi Shan, Qingfeng Ma, Jie Lu

**Affiliations:** ^1^Department of Radiology and Nuclear Medicine, Xuanwu Hospital, Capital Medical University, Beijing, China; ^2^Beijing Key Laboratory of Magnetic Resonance Imaging and Brain Informatics, Beijing, China; ^3^Department of Neurology, Xuanwu Hospital, Capital Medical University, Beijing, China

## Abstract

*Purpos*e. We investigated the disparate influence of lesion location on functional damage and reorganization of the sensorimotor brain network in patients with thalamic infarction and pontine infarction. *Methods*. Fourteen patients with unilateral infarction of the thalamus and 14 patients with unilateral infarction of the pons underwent longitudinal fMRI measurements and motor functional assessment five times during a 6-month period (<7 days, at 2 weeks, 1 month, 3 months, and 6 months after stroke onset). Twenty-five age- and sex-matched controls underwent MRI examination across five consecutive time points in 6 months. Functional images from patients with left hemisphere lesions were first flipped from the left to the right side. The voxel-wise connectivity analyses between the reference time course of each ROI (the contralateral dorsal lateral putamen (dl-putamen), pons, ventral anterior (VA), and ventral lateral (VL) nuclei of the thalamus) and the time course of each voxel in the sensorimotor area were performed for all five measurements. One-way ANOVA was used to identify between-group differences in functional connectivity (FC) at baseline stage (<7 days after stroke onset), with infarction volume included as a nuisance variable. The family-wise error (FWE) method was used to account for multiple comparison issues using SPM software. Post hoc repeated-measure ANOVA was applied to examine longitudinal FC reorganization. *Results*. At baseline stage, significant differences were detected between the contralateral VA and ipsilateral postcentral gyrus (cl_VA-ip_postcentral), contralateral VL and ipsilateral precentral gyrus (cl_VL-ip_precentral). Repeated measures ANOVA revealed that the FC change of cl_VA-ip_postcentral differ significantly among the three groups over time. The significant changes of FC between cl_VA and ip_postcentral at different time points in the thalamic infarction group showed that compared with 7 days after stroke onset, there was significantly increased FC of cl_VA-ip_postcentral at 1 month, 3 months, and 6 months after stroke onset. *Conclusions*. The different patterns of sensorimotor functional damage and reorganization in patients with pontine infarction and thalamic infarction may provide insights into the neural mechanisms underlying functional recovery after stroke.

## 1. Introduction

Motor function impairment, as well as rehabilitation, depends highly on infarction locations in patients with stroke [1, 2]. A previous structural neuroimaging study revealed that patients with basal ganglia infarction displayed structural impairment in the ipsilesional sensorimotor cortex, whereas patients with pontine stroke mainly displayed cerebellar damage as well as structural restructuring in the precuneus [3], indicating that different types of brain damage and reorganization might be caused by location-specific lesions in patients with stroke [4]. However, the neural mechanisms underlying brain functional connectivity changes caused by distinct stroke locations remain poorly understood.

Based on the anatomical site of cerebral infarction, patients can be subdivided into supratentorial and infratentorial cerebral infarctions. Most supratentorial infarction-associated functional magnetic resonance imaging (fMRI) studies have focused on stroke patients with lesions in the basal ganglia or corona radiata [5, 6], yet overlooked thalamic infarctions. The thalamus has been the focus of investigation in neuroscience, as nearly all incoming information to the cortex is routed through this brain area [7]. Pontine regions are common sites of infratentorial cerebral infarction [8] and involve the motor pathway. Through the ventral anterior (VA) and ventral lateral nuclei (VL) of the thalamus, information processing in the basal ganglia (putamen and caudate) returns to the cerebral cortex, and minor output from the basal ganglia descends to the brain stem in the cortico-basal ganglia loop [9]. Thalamic and pontine infarctions may affect sensorimotor function differently.

Previous evidence suggests that thalamic and pontine infarction could cause impairment of functional connections in brain regions outside of the lesion [10, 11]. However, it is not clear whether patients with thalamic infarction and patients with pontine infarction display different characteristics of functional connectivity involving the sensorimotor brain areas. Thus, we retrospectively analyzed a longitudinal dataset in which we collected data for patients with stroke with thalamic lesions, pontine lesions, and healthy controls. We hypothesized that thalamic infarction and pontine infarction patients experience different patterns of sensorimotor functional damage and reorganization.

## 2. Methods

### 2.1. Participants

Twenty-eight right-handed stroke patients with different degrees of neurological dysfunction were recruited from inpatient services at Xuanwu Hospital of Capital Medical University (Beijing, China). All participants provided written informed consent prior to assessment. The inclusion criteria were as follows: (1) first-ever ischemic stroke (within 7 days of symptom onset), (2) unilateral lesions involving pons or thalamus were confirmed by diffusion-weighted imaging (DWI), and (3) age 18 to 75 years old. Exclusion criteria were as follows: (1) unclear onset time, (2) lesions outside the pons or thalamus, (3) recurrence of infarction or secondary hemorrhage during follow-up, and (4) deafness and/or blindness, or aphasia that might prevent completion of the study. Fourteen patients with unilateral infarction of the thalamus (TI group) and 14 patients with unilateral infarction of the pons (PI group) were enrolled in the current study. Twenty-five age-and sex-matched healthy control participants were included as the normal control group (NC group).

### 2.2. Study Protocol

**Figure 1:**
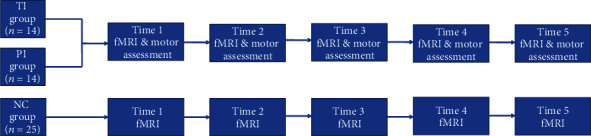
Study protocol. TI: thalamic infarction, PI: pontine infarction: NC: normal control, fMRI: functional magnetic resonance imaging, Time 1: within 7 days after stroke onset, Time 2: 2 weeks after stroke onset, Time 3: 1 month after stroke onset, Time 4: 3 months after stroke onset, Time 5: 6 months after stroke onset.

The current study protocol was planned as a 6-month longitudinal design, during which patients with stroke underwent assessment using the Fugl-Meyer (FM) scale and magnetic resonance imaging (MRI). Those data were collected five times after infarction occurred, during the first week after symptom onset (<7 days), 2 weeks, 1 month, 3 months, and 6 months after stroke onset. We then acquired MRI data on day 0 (baseline), 2 weeks, 1 month, 3 months, and 6 months for the normal controls (Figure 1).

### 2.3. Image Acquisition

All participants (patients with stroke and healthy volunteers) were invited to participate in 5 imaging sessions. MRI data were acquired using a 3T MR scanner equipped with a 12-channel coil (TimTrio, Siemens AG, Erlangen, Germany). Full brain structural images were collected using a sagittal 3D-magnetization-prepared rapid acquisition gradient echo (3D-MPRAGE) T1-weighted sequence with the following parameters: TR/TE = 1600/2.15 msec, flip angle = 9°, FOV = 256 mm × 256 mm, matrix size = 256 × 256, and voxel size = 1 × 1 × 1 mm^3^. Resting-state functional MRI (fMRI) data were obtained using a gradient-echo echo-planar imaging sequence with the following parameters: TR/TE = 3000/30 msec, flip angle = 90°, voxel size = 3 × 3 × 3 mm^3^, matrix size = 64 × 64, gap = 0 mm, number of slices = 43, and 124 time points. During fMRI scanning, all participants were instructed to remain motionless, stay awake, and keep their eyes open. Axial fast spin-echo T2-weighted, fluid attenuation inversion-recovery, and DWI examinations were also performed.

#### 2.3.1. Behavioral Assessment

The degree of motor deficit was assessed independently by two neurologists on the same day as the MRI data acquisition. The two scores were averaged to provide an estimate. Thirty-three tasks in the FM scale were used to evaluate patients' motor function, limb coordination, and active joint function of the upper limbs [12, 13]. Each score was given on a scale of 0 to 2, according to the patient's response to the specific task (0 = patient was unable to perform the task, 1 = patient could partially perform the task, and 2 = patient could accomplish the task), thus, the maximum possible total score was 66. All scores of the total 33 tasks were summed and normalized to a score between 0 and 100 as the following formula:

(1) $$\begin{array}{c} \text{The normalized FM score}=\frac{\text{the sum of the }33\text{ scores}}{66}\times 100\%. \end{array}$$

### 2.4. Normalized Infarction Volume Measurement

Measurement of infarction lesion volumes of PI and TI was performed manually using MRIcron software (version 1.40), including the following steps: (1) infarction volume measurement: two experienced physicians individually measured the infarction size using DWI images for patients during the subacute stage and fluid-attenuated inversion recovery (FLAIR) images during the chronic stage. The scores measured by the two physicians were then averaged. (2) Normalized infarction volume measurement: the infarction volume of patients was normalized to reduce the individual differences in brain volume. The median sagittal plane area was measured in the 3D-MPRAGE image [14], which is highly correlated with the volume of the brain (correlation coefficient = 0.98) [15]. The following formula was used to standardize the infarction volume of the TI and PI groups:

(2) $$\begin{array}{c} \text{normalized cerebral infarction volume}=\frac{\text{infarciton volume}\times \text{average area of the median sagital plane}}{\text{area of median sagittal plane of stroke patients}}. \end{array}$$

### 2.5. Resting-State fMRI Data Preprocessing

Resting-state fMRI data were preprocessed using data processing and analysis of brain imaging (DPABI) software [16] and SPM12 toolbox (http://www.fil.ion.ucl.ac.uk/spm/software/spm12). The following steps were performed: (1) discard the first 10 EPI volumes, (2) slice timing correction, (3) head motion correction, (4) spatial normalization to MNI space using an EPI template with a voxel size of 3 mm × 3 mm × 3 mm by DARTEL, (5) data were smoothed using a Gaussian kernel of 6 mm full width at half maximum (FWHM), (6) linear regression was performed to remove the effects of the white matter and cerebrospinal fluid by 99% mask, (7) band-pass filtering between 0.01-0.1 Hz, and (8) preventing focal infarct tissue from affecting the algorithm, the imaging data from the stroke patients with lesions in the left hemisphere were flipped from left to right along the median sagittal line. The right hemisphere was defined the ipsilesional side, and the left hemisphere was defined as the contralesional side in all patients with stroke.

### 2.6. Regions of Interest (ROIs) Definition and Functional Connectivity Calculations

**Figure 2:**
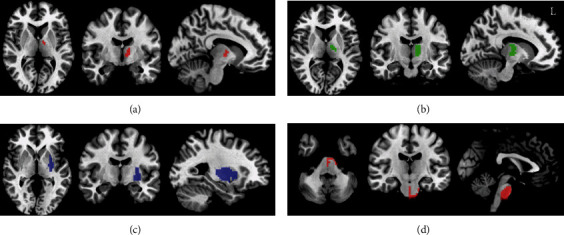
The four ROIs in the contralesional VA nuclei of thalamus (a), contralesional VL nuclei of thalamus (b), contralesional dorsal lateral putamen (c), and pons (d). L: left.

Subcortical areas, such as the basal ganglia (putamen), thalamus, cerebellum, and brainstem nuclei, are important components of the motor network [17]. They have direct or indirect structural connections with the sensorimotor cortex. According to previous, cross-sectional studies, FC changes between the brain regions of the putamen, thalamus, and sensorimotor brain areas were associated with patients with stroke [11, 18]. We defined the dorsal lateral putamen (dl-putamen) [19] and VA and VL nuclei of the thalamus [20] as seed ROIs. Meanwhile, the pons [21] was also defined as another ROI to examine whether the ROI adjacent to the lesion would affect the FC in the PI group. All ROIs are shown in Figure 2. After flipping the infarction lesion from left to right along the midline for patients with left hemispheric lesions, the left side corresponded to the contralesional hemisphere. Therefore, ROIs were extracted from the healthy hemisphere in patients with stroke.

The voxel-wise functional connectivity analyses between the reference time course of each ROI (the dl-putamen, VA, and VL nuclei of the thalamus and pons) and the time course of each voxel in the brain areas were performed to generate seed-based FC maps at baseline stage, 2weeks, 1 month, 3 months, and 6 months after stroke. Pearson's correlation coefficients between the average time series of the ROIs and sensorimotor brain areas were computed to obtain seed-based FC maps. For group analyses, the correlation coefficients were transformed to *z* values using Fisher's *z*-transformation to improve the normality of the correlation coefficient.

### 2.7. Statistical Analyses

#### 2.7.1. Demographic and Clinical Data Analyses

Statistical analyses of demographic and clinical data were conducted using SPSS 17.0. The statistical significance threshold was set at *p* < 0.05. A Chi-square test was used to identify differences in sex and handedness among the PI, TI, and NC groups. For clinical variables, the differences in age, normalized infarction volume, and FM scores were analyzed by one-way ANOVA in the PI and TI groups.

#### 2.7.2. Functional MRI Analyses

The infarction volume was the largest within 7 days of the onset of ischemic stroke compared to the follow-up time points. Therefore, we considered that larger infarct volumes would indicate more significant effects of infarct volume on FC in patients with stroke. The FC in patients with stroke (PI and TI groups) differed the most from normal controls at baseline stage. One-way ANOVA controlling for infarction volume of patients with stroke (the infarction volume of normal controls was defined as 0) was used to identify group differences of FC among PI, TI, and NC groups at baseline stage. The peak voxel of the corresponding sensorimotor area (i.e., precentral and postcentral gyrus) of automated the anatomical labeling (AAL) [22] mask was used for small volume correction (SVC). The SVC is a family wise error multiple correction method provided by SPM, which has been widely used in neuroimaging studies [23]. Then, sphere ROIs with a 5 mm radius located at the peak voxel were built for post hoc analysis. The mean FC values from the sphere ROIs were extracted for further analyses.

Next, a post hoc “5 (time) ×3 (group)” repeated-measure ANOVA model was established to explore the differences in longitudinal FC changes among the three groups. The changes in FC in different brain regions among the three groups over a long-term follow-up of 6 months were examined for interaction effects of “time” by “group.” The significance threshold was set at *p* < 0.05.

## 3. Results

### 3.1. Demographic and Clinical Parameters

Detailed demographic and clinical findings for the PI, TI, and NC groups are provided in Table 1. No significant differences were observed among the PI, TI, and NC groups in terms of age (*p* = 0.103, one-way ANOVA), sex (*p* = 0.719, chi-square test), or handedness (*p* = 0.341, chi-square test). The normalized infarction volume decreased significantly during the observation period in the PI (*F*_(4, 65)_ = 4.77, *p* = 0.002) and TI groups (*F*_(4, 65)_ = 7.16; *p* ≤ 0.001). Longitudinal FM examination revealed significant improvement over time in the PI (*F*_(4, 65)_ = 8.92, *p* ≤ 0.001) and TI groups (*F*_(4, 65)_ = 4.94, *p* = 0.002).

### 3.2. Comparison of Seed-Based FC among PI, TI, and NC Groups within 7 Days after Stroke

**Figure 3:**
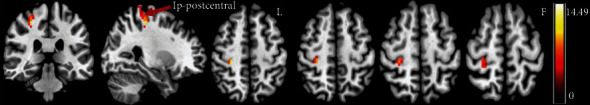
One-way ANOVA revealed a significant difference in FC between the contralateral VA and ipsilateral postcentral gyrus (cl_VA-ip_postcentral) among three groups at baseline stage. L: left, Ip: ipsilateral postcentral gyrus, *p*_uncorrected_ < 0.001.

**Figure 4:**
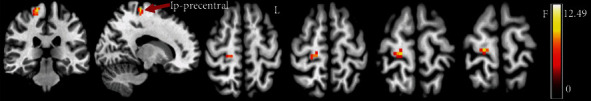
One-way ANOVA displayed a significant difference in FC between the contralateral VL and ipsilateral precentral gyrus (cl_VL-ip_precentral) among three groups at baseline stage. L: left, Ip: ipsilateral postcentral gyrus; *p*_uncorrected_ < 0.001.

At baseline stage (within 7 days after stroke), one-way ANOVA analysis of FC between seed-based (the contralesional side of VA, VL, pon, and putamen) and whole-brain regions indicated a significant difference in FC between the contralateral VA and ipsilateral postcentral gyrus (cl_VA-ip_postcentral) (peak *F*_(2, 49)_ = 14.49, *p*_FWE_ = 0.043, MNI: 27, -30, 54) among the three groups (Figure 3). The sensorimotor-related brain regions with significant differences in functional connectivity were also observed in the ipsilateral precentral gyrus with contralateral VL (cl_VL-ip_precentral) (peak *F*_(2, 49)_ = 12.49, *p*_FWE_ = 0.037, MNI: 15, -27, 69) among the three groups (Figure 4). However, with the contralateral pon (cl_pon) and contralateral putamen (cl_putamen) as the seed-ROI, one-way ANOVA analysis of the FC indicated no significant differences in sensorimotor-related brain regions among the three groups. Post hoc comparisons of the baseline stage in the three groups showed significant difference of cl_VA-ip_postcentral FC between TI and both normal control (*t* = 5.057, *p* < 0.001) and PI groups (*t* = 3.024, *p* = 0.006). However, there was no significant difference in FC of cl_VA-ip_postcentral between normal control and PI groups (*t* = 1.592, *p* = 0.12). Meanwhile, the post hoc comparisons of the baseline stage among the three groups showed significant difference of cl_VL-ip_precentral FC between PI and both normal control (*t* = 5.08, *p* < 0.001) and TI groups (*t* = 5.01, *p* < 0.001). However, there was no significant difference in FC of cl_VL-ip_precentral between normal control and TI groups (*t* = 1.592, *p* = 0.12).

### 3.3. Longitudinal FC Analysis among the TI, PI, and NC Groups

**Figure 5:**
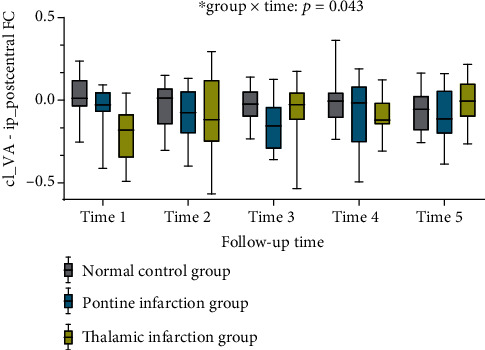
Longitudinal cl_VA-ip_postcentral FC changes among three groups.

We further explored longitudinal FC changes during the follow-up period in the TI, PI, and NC groups by extracting FC values in cl_VA-ip_postcentral, cl_VL-ip_precentral, cl_pon-ip_postcentral, cl_pon-ip_precentral, cl_putamen-ip_postcentral, and cl_putamen-ip_precentral. No significant differences in “time” main effect were detected for cl_VA-ip_postcentral (repeated measures ANOVA: *F*_(4,200)_ = 0.220, *p* = 0.927). The “group” main effect of cl_VA-ip_postcentral differed significantly among the three groups (*F*_(2, 50)_ = 7.193, *p* = 0.002). There was a significant “group×time” interaction effect of cl_VA-ip_postcentral (repeated measures ANOVA: *F*_(8,200)_ = 2.702, *p* = 0.008, Figure 5). Moreover, there was no significant difference in “time” (repeated-measure ANOVA: *p* = 0.282) or “group” (repeated-measure ANOVA: *p* = 0.314) main effect of cl_VL-ip_precentral, cl_pon-ip_postcentral, cl_pon-ip_precentral, cl_putamen-ip_postcentral, and cl_putamen-ip_precentral. The interaction effect of FC changes of cl_VL-ip_precentral, cl_pon-ip_postcentral, cl_pon-ip_precentral, cl_putamen-ip_postcentral, and cl_putamen-ip_precentral did not differ significantly among the three groups over time (*p* > 0.504).

Results of the plot of the study indicated that the interaction effect results were mainly detected one month ago; hence, we considered one month as the cut-off point and divided the follow-up time of the study into two stages. A “time×group” repeated-measure ANOVA indicated a significant interaction effect of cl_VA-ip_postcentral that varied significantly among three groups at one month after stroke, *F*_(4,100)_ = 2.980, *p* = 0.023. However, the “time×group” repeated-measure ANOVA failed to detect a significant interaction effect among the three groups from 1 month to 6 months after stroke, *F*_(4,100)_ = 1.523, *p* = 0.201.

Repeated measurement analysis was used to detect changes in FC within each group, and the results showed the FC value of cl_VA-ip_postcentral with no significant difference between the PI and NC groups. However, the FC value of cl_VA-ip_postcentral was significantly different in the TI group. Compared with 7 days after stroke onset, there was a significant increase in FC of cl_VA-ip_postcentral at 1 month (*T*_(13)_ = 2.550, *p* = 0.024), 3 months (*T*_(13)_ = 2.859, *p* = 0.013), and 6 months (*T*_(13)_ = 3.178, *p* = 0.007) after stroke onset in the TI group, and the difference in FC in cl_VA-ip_postcentral between two time points in the TI group became increasingly greater with the prolongation of time. Compared with 7 days after stroke onset, there was no significant increase in FC of cl_VA-ip_postcentral in the TI group at 2 weeks after stroke onset (*p* = 0.115).

### 3.4. Heterogeneity in PI Group

**Figure 6:**
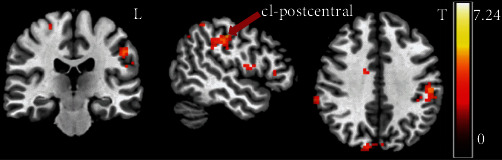
*T* test of FC between the contralateral postcentral gyrus with contralateral VA (cl_VA-cl_ postcentral) between PI1 and PI2 at baseline stage (within 7 days after stroke). L: left, *p*_uncorrected_ < 0.005.

**Figure 7:**
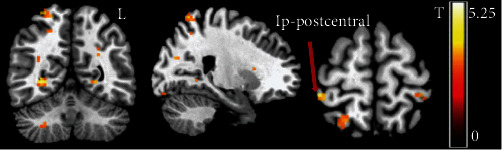
*T* test of FC between the ipsilateral postcentral gyrus with contralateral VL (cl_VL-ip_postcentral) between PI1 and PI2 at baseline stage (within 7 days after stroke). L: left, *p*_uncorrected_ < 0.005.

The large differences in infarction lesion volume may have resulted in greater heterogeneity in the functional connectivity of patients in PI group (the range of infarction volume is 2.44 ml~ 23.2 ml); therefore, we suspected that heterogeneity of infarction lesion volume may have affected FC changes in the PI group. To confirm our hypothesis, the pontine infarction group was further divided into two subgroups according to the infarction volume at baseline. Patients with infarction volume < 10 ml were assigned to the PI1 group (7 patients) and the rest (>10 ml) were assigned to the PI2 group (7 patients). A *t*-test of FC between seed-based (the contralateral VA and VL) and whole-brain regions between the PI1 and PI2 groups indicated significant differences in FC of cl_VA-cl_postcentral (peak *T* = 4.8, *p*_uncorrected_ < 0.001, Figure 6), and cl_VL-ip_postcentral (peak *T* = 4.5, *p*_uncorrected_ < 0.001, Figure 7). There was a significant difference in the brain area of the sensorimotor cortex between the PI1 and PI2 groups. Therefore, the greater heterogeneity of infarct volume in patients with pontine infarction resulted in no differences in FC among the PI, TI, and NC groups.

### 3.5. Correlation Analysis in the TI Group

The brain region of the cl_VA-ip_postcentral functional connectivity at baseline was not significantly correlated with motor improvement, as measured by the FM scale at 6 months after onset (*r* = 0.104, *p* = 0.724). Pearson correlation analyses indicated that the FC changes (FC changes = FC_Time5_ − FC_Time1_) of cl_VA-ip_postcentral_postcentral were not correlated with the changes in the FM scale (FM changes = FM_Time5_ − FM_Time1_) in the TI group (*r* = −0.179, *p* = 0.540). The correlation between infarct volume and FM was also analyzed in the TI group. No significant correlation was observed between infarction volume at baseline stage and FM at 6 months after stroke onset (*r* = −0.366, *p* = 0.198). Infarction volume changes (infarction volume changes = infarction volume_Time5_–infarction volume_Time1_) did not correlate with the changes in the FM scale (FM changes = FM_Time5_ − FM_Time1_) in the TI group (*r* = −0.305, *p* = 0.289).

## 4. Discussion

In the current study, we investigated the longitudinal changes in resting-state FC between patients with TI, patients with PI, and healthy participants during a 6-month, poststroke follow-up period. At the baseline stage, our results demonstrated significant differences in FC between the contralateral VA and ipsilateral postcentral gyrus, contralateral VL, and ipsilateral precentral gyrus among the three groups. However, further analysis of longitudinal FC changes revealed a significant difference in FC between cl_VA and ip_postcentral during the follow-up period among the three groups. The TI group displayed significantly decreased FC in cl_VA-ip_postcentral at the baseline stage compared with the PI and NC groups and then significantly increased FC at 1, 3, and 6 months after stroke onset.

Resting-state functional connectivity can reflect interactions between two remote regions. In a previous resting-state study, decreased connectivity between the bilateral M1 was demonstrated in patients with stroke with right hemispheric subcortical infarcts within 6 months after stroke onset [24]. In the current study, FC of the ipsilateral postcentral to contralateral VA was significantly decreased between the TI and PI and NC groups within 7 days after stroke. Recently, Golestani et al. [6] detected a decrease in resting-state functional connectivity between the ipsilesional sensorimotor cortex and subcortical regions, namely, the bilateral caudate and thalamus in patients with chronic stroke, and these changes could be detected within hours poststroke. Our results suggest that the infarct lesion disrupted the anatomical connections between two remote regions, which may further result in reduced functional connectivity between the ipsilesional sensorimotor cortex and contralesional normal brain in patients with stroke.

The ventral anterior and ventral lateral nuclei of the thalamus are generally referred to as the “motor thalamus.” The VA and VL of the thalamus serve as relay stations for incoming information from external sources to the sensorimotor cortex [25]. Previous research indicates had showed abnormal fiber integrity [26], decreased ReHo [27], and decreased FC [11] in brain regions related to sensorimotor motor circuits in patients with TI. He et al. reported decreased FC in the ipsilesional posterior central gyrus in patients with TI presenting with somatosensory deficits, which is related to somatosensory dysfunction in patients with TI [11]. Lower thalamo-postcentral connectivity may represent decreased sensorimotor integration [28]. In the current study, we demonstrated decreased FC between the ipsilateral postcentral and contralateral VA in the TI group at the baseline stage, which is highly consistent with previous findings. The reduced FC implies the breakdown of the harmonious interaction between the ipsilateral sensorimotor and contralateral thalamus after thalamic stroke.

Another interesting finding of the current study is a gradual increase in FC between cl_VA and ip_postcentral was observed from 2 weeks to 6 months after stroke onset. Compared with within the 7th day after stroke onset, the change in FC between the cl_VA and ip_postcentral increased significantly at 1, 3, and 6 months after stroke onset in patients with thalamic infarction. A previous study investigated a similar trend of FC changes, which decreased at the acute stage of stroke and then gradually increased thereafter [29]. More severe symptoms were associated with decreased functional connectivity, and recovery of behavioral function was associated with increased functional connectivity [30, 31]. We considered that the reorganization of FC between cl_VA and ip_postcentral contributed to the recovery of sensorimotor function in our participants. In patients with sufficient integrity of the ipsilesional sensorimotor cortex and its corticospinal tract, motor recovery may occur rapidly after stroke and is mediated by the reacquisition of normal dominance by the ipsilesional sensorimotor cortex. FC between the ipsilesional sensorimotor areas and bilateral thalamus in patients with TI, which demonstrates that there are changes in functional connectivity associated with recovery, is increased in patients with TI presenting with somatosensory deficits [11]. The increased FC in the sensorimotor network or ipsilesional motor cortex may be related to gradual disappearance of diaschisis [32] or axonal sprouting [33] to establish new connections, and motor recovery is related to the normalization of its reinstatement [11, 34, 35].

However, we did not detect a significant correlation between functional connectivity in the cl_VA-ip_postcentral and FM scales in the TI group. Patients with TI with mild motor deficits and a small sample size in this study have caused this result. Another probable cause was that we evaluated patients' motor function of the upper limbs (i.e., reflex activity and flexor synergy). Nevertheless, the postcentral gyrus is also involved in sensory processing, and the decreased activation in the postcentral cortex is indicative of attenuated sensory processing [28, 36]. Although we did not observe a significant correlation between the infarct volume at onset and FM scores at 6 months in the TI group, the relatively strong correlation coefficient may indicate that a significant correlation between the infarct volume and FM scores may be detectable with a larger sample of the TI group.

In our study, there were no significant differences in FC changes between ROIs and the sensorimotor cortex in patients with PI. Wei et al. [29] explored longitudinal FC alterations during the follow-up period in patients with the left pontine infarction (LPI) and right pontine infarction (RPI) groups, using brain regions with altered cerebral blood flow (CBF) in longitudinal analysis as seed-ROIs. In the RPI group, there were no brain regions with significant longitudinal differences among the four time points. As our patients with pontine infarction had both left and right lesions, we investigated the intrapontine group differences in functional connectivity induced by infarction lesion size. We observed significant differences in functional connectivity between cl_VA and cl_ postcentral as well as between cl_VL and ip_postcentral. We considered that the heterogeneity within the pontine group might be associated with nonsignificant results during intergroup comparisons.

## 5. Conclusions

In this study, we analyzed the FC changes in sensorimotor brain areas in patients with PI versus those with TI during follow-up of 6 months. The main findings were that FC significantly decreased between cl_VA and ip_postcentral in patients with TI at baseline as compared with the PI and NC groups, and the TI group exhibited gradual increases in FC between cl_VA and ip_postcentral thereafter. Therefore, FC increase between cl_VA and ip_postcentral suggests that the sensorimotor brain area may be responsible for the recovery of motor function in patients with thalamic stroke. Additionally, we did not detect any significant differences in FC changes between ROIs and the sensorimotor cortex in patients with PI. Heterogeneity within the pontine group may be associated with nonsignificant results during the intergroup comparisons.

### 5.1. Limitations

There are a few limitations to consider. One of the weaknesses of our pilot study was the lack of data regarding assessment of other functions, for example, sensory and cognitive function. Future studies should address the sensory and cognitive function changes in patients with thalamic infarction and pontine infarction. Second, a limited number of cases due to the rarity of isolated unilateral thalamic strokes and isolated unilateral pontine strokes may have prevented us from providing conclusive evidence for FC changes in patients with thalamic infarction and pontine infarction. We expect to expand the sample size in our future work to further elucidate our findings.

## Figures and Tables

**Table 1:** Demographic and clinical information of all participants.

Characteristics	NC (*n* = 25)	PI (*n* = 14)	TI (*n* = 14)	*p* value
Age (years)	51.57 ± 10.82	58.00 ± 6.73	51.57 ± 10.82	0.103^a^
Sex (F/M)	10/15	4/10	5/9	0.719^b^
Handedness (L/R)	1/24	0/14	0/14	0.341^b^
Lesion side (L/R)	——	5/9	11/3	——
Normalized lesion volume (ml)	——			*F*_PI_ = 4.77; *p* = 0.002^∗^ *F*_TI_ = 7.16; *p* ≤ 0.001^∗^
Time 1		12.72 ± 7.34	0.78 ± 0.55	
Time 2		9.68 ± 6.74	0.49 ± 0.38	
Time 3		6.05 ± 4.55	0.34 ± 0.28	
Time 4		5.43 ± 3.66	0.22 ± 0.18	
Time 5		5.43 ± 4.53	0.18 ± 0.17	
FM-upper (0-100)				*F*_PI_ = 8.92; *p* ≤ 0.001^∗^ *F*_TI_ = 4.94; *p* = 0.002^∗^
Time 1		48.92 ± 33.82	86.15 ± 16.69	
Time 2		67.21 ± 28.78	91.88 ± 9.07	
Time 3		82.58 ± 18.93	96.6 ± 5.11	
Time 4		88.74 ± 13.38	97.95 ± 3.24	
Time 5		93.45 ± 0.09	99.03 ± 1.94	

Data are presented as mean ± SD. ^a^One-way analysis of covariance (ANCOVA), ^b^Chi-square test. Abbreviations: NC: normal control, PI: pontine infarction, TI: thalamic infarction, F: female, M: male, L: left, R: right, Time 1, Time 2, Time 3, and Time 4, and Time 5 different time subgroups from 1 week to 6 months after stroke, SD: standard deviation, ^∗^*p* < 0.05.

## Data Availability

Datasets analyzed during the current study are available from the corresponding author on reasonable request.
